# Case Report: Long-term complete response to PSMA-targeted radioligand therapy and abiraterone in a metastatic prostate cancer patient

**DOI:** 10.3389/fonc.2023.1192792

**Published:** 2023-04-28

**Authors:** David Parker, Jessica Zambelli, Montana Kay Lara, Trevor Hamilton Wolf, Amber McDonald, Erica Lee, Lotfi Abou-Elkacem, Eva J. Gordon, Richard P. Baum

**Affiliations:** ^1^ Private Health Management, Los Angeles, CA, United States; ^2^ Curanosticum, Frankfurt-Wiesbaden, Germany

**Keywords:** case report, PSMA, radioligand therapy, prostate cancer, metastatic castration resistant prostate cancer, Lu-PSMA-617, theranostics

## Abstract

Despite decades of research and clinical trials, metastatic castration-resistant prostate cancer (mCRPC) remains incurable and typically fatal. Current treatments may provide modest increases in progression-free survival but can come with significant adverse effects and are disaggregated from the diagnostic imaging needed to fully assess the spread of metastatic disease. A theranostic approach, using radiolabeled ligands that target the cell surface protein PSMA, simplifies the visualization and disease treatment process by enabling both to use similar agents. Here, we describe an exemplary case wherein a gentleman in his 70s with mCRPC on diagnosis was treated with ^177^Lu–PSMA-617 and abiraterone, and remains disease-free to date, over five years later.

## 1 Introduction

The increasing cellular and molecular resolution of diagnostic tools for human disease reinforces the need for targeted, multimodal therapeutic approaches. Theranostics, the simultaneous use of diagnostics and therapeutics, leverages the molecular strengths of both. One implementation of theranostics utilizes non-toxic radiolabeling of diseased tissue combined with targeted radioactive drugs for treatment. Some of these treatments also have utility as imaging tools to monitor tracer uptake and guide treatment decisions. Originally pioneered in the 1940s using radioactive iodine for characterizing and treating thyroid cancer, theranostics has recently been applied to metastatic castration-resistant prostate cancer (mCRPC), among other cancers, with great success (1–5).

Indeed, on December 1, 2020, and March 23, 2022, the US Food and Drug Administration (FDA) approved ^68^Ga–PSMA-11 and ^177^Lu–PSMA-617 for imaging and treating mCRPC, respectively. These agents bind to prostate-specific membrane antigen (PSMA), a protein prevalent on the surface of prostate cancer cells, either enabling visualization or delivering toxic radiation, depending on the radioactive element employed (6). This approach takes advantage of the simultaneous diagnostic and therapeutic power of PSMA labeling.

Standard care for localized prostate cancer involves expectant management and monitoring for progression, followed by surgery and radiation. Upon metastasis, the first line of treatment is androgen deprivation therapy (ADT). However, metastatic prostate cancer can be unresponsive to ADT, termed castration-resistant, either at treatment outset or after initial sensitivity. To treat patients who are unresponsive to ADT, there have been several treatment options designed to re-sensitize patients to ADT or disrupt the androgen axis (7–9), in addition to therapeutic strategies such as systemic chemotherapy with taxane-based agents or radium-223 to mitigate bony metastases, the most common metastatic site in prostate cancer (10, 11). Nevertheless, PSMA-targeted radioligand therapy improves upon current strategies because unlike ADT, resistance to ^177^Lu–PSMA-617 does not readily emerge. Instead, PSMA expression increases with prostate tumor malignancy and metastases, potentially yielding greater efficacy from ^177^Lu–PSMA-617 (12, 13).

Here, we present an exemplary case wherein a 73-year-old male was treated for mCRPC in 2017 with ^177^Lu–PSMA-617. After progressing through initial standard of care treatment with chemotherapy and ADT, the patient underwent a gallium-68 (Ga-68) PSMA PET/CT scan revealing widespread, PSMA-expressing bony metastases. Remarkably, after four treatments with PSMA-targeting radioligand therapy over the course of ten months, while continuing abiraterone treatment, the patient continues to have no evidence of disease as of the writing of this article, over five years after initiating PSMA therapy.

## 2 Case description

**Figure 1:**
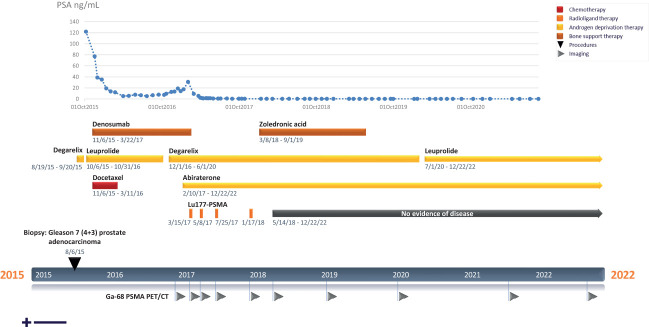
Clinical timeline.

We report a case of a now 80-year-old male who presented in May 2015 at a routine screening with highly elevated prostate specific antigen (PSA). Eight months prior, the patient had initiated testosterone therapy with Androgel to treat hypogonadism and fatigue. After discontinuing testosterone, repeat screening showed PSA levels remained elevated. In August 2015, a prostate core biopsy revealed prostatic adenocarcinoma originally classified as Gleason 7 (3 + 4). CT and bone scan revealed extensive lymph and bone metastases throughout the axial and appendicular skeleton. The patient began ADT with degarelix to quickly lower testosterone and PSA levels (**Figure 1**).

A second pathology review in September 2015 upstaged the patient to Gleason 7 (4 + 3), stage 4B, which has a five-year survival rate of ~30% (14). In October 2015, ADT was switched to leuprolide, and in the following month he began denosumab for bone support. Starting in November 2015, the patient underwent 6 cycles of chemotherapy with docetaxel, based on evidence from the CHARRTED trial, in which addition of docetaxel at the beginning of treatment with ADT was demonstrated to improve overall survival in men with metastatic prostate cancer (15–17). A post-chemotherapy CT and bone scan in March 2016 showed improved appearance of known bone metastases suggesting some degree of treatment response and no evidence of solid organ metastases or abdominopelvic lymphadenopathy. PSA was also well-controlled on ADT, and the patient continued with leuprolide therapy alone.

A rise in PSA level in October 2016 prompted a switch to ADT with degarelix as the patient’s testosterone was still above castrate level. In December, 2016, multiple imaging studies (CT, MRI, and 18-F bone imaging PET/CT) showed continued osteoblastic skeletal metastasis. At this time, the patient was considering multiple clinical trials and underwent a gallium-68 (Ga-68) PSMA PET/CT scan to evaluate his candidacy for PSMA-targeting radioligand therapy. The scan showed multiple PSMA avid lesions consistent with active bone metastasis and no evidence of PSMA avidity in soft tissue, confirming his candidacy for lutetium-177 (Lu-177) PSMA radioligand therapy. Moreover, persistent castration-resistant disease supported the addition of abiraterone, which was initiated in February 2017.

**Figure 2:**
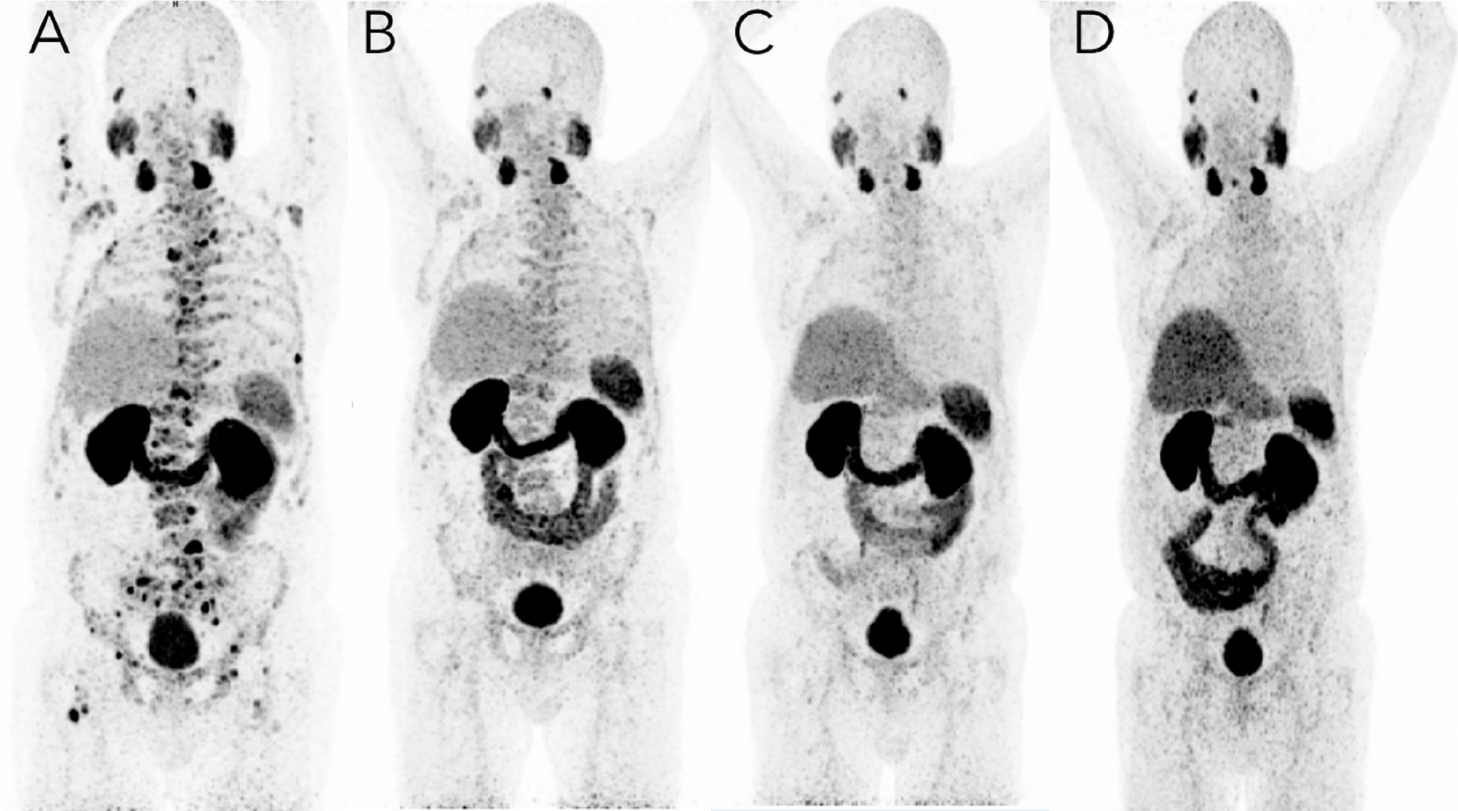
PSMA imaging before and after targeted radioligand therapy with ^177^Lu–PSMA-617. A 73-year-old male with mCRPC in 2017 with multiple bone and lymph node metastases with increased PSMA expression. **(A)** Imaging on March 14, 2017, just before the first dose of [^177^Lu]–PSMA-617 therapy; the baseline coronal PET [^68^Ga]-PSMA-11 PET maximum intensity projection (MIP) image shows bone metastases with significantly higher PSMA expression as well as the normal biodistribution of the tracer. **(B)** Imaging on July 24, 2017, just before the third dose of Lu-177 PSMA radioligand therapy, showed intense but decreasing uptake with an excellent target-to-background ratio, corresponding with molecular response to RLT therapy. Imaging four months (May 15, 2018) **(C)** and just over one year (February 11, 2019) **(D)** after the fourth dose of [^177^Lu]–PSMA-617, the [^68^Ga]-PSMA-11 PET MIP image shows multiple decreased or disappeared metastases. No evidence of PSMA-positive lymph nodes or new metastases and very mild uptake was noted in the central skeleton and sternum. Overall, no new and no intense PSMA-avid lesions were noted.

At this time, ^177^Lu–PSMA-617 was not approved in the U.S., so this patient travelled to Germany to receive treatment. While continuing with abiraterone, the patient completed four cycles of ^177^Lu–PSMA-617 therapy over the course of 10 months (due to the patient’s robust response, the interval between the 3^rd^ and 4^th^ cycles was extended) and tolerated treatment well without any adverse effects (**Figure 1**). Whole-body planar imaging (4, 24, 48, and 72 hours after radioligand therapy) and SPECT/CT (24 and 48 hours after radioligand therapy) were performed to monitor treatment uptake. Interval and follow-up scans showed continued response to treatment with near complete regression of disease and dramatic decrease of PSMA expression in the bone (**Figure 2**; **Table 1**). The patient continues on leuprolide every three months along with daily abiraterone, and remains stable as of the time of this publication, over five years after his last treatment in January, 2018.

**Table 1:** PSMA imaging interval and follow-up results.

Date	Findings
30AUG2021	Extremely faint, inhomogeneous PSMA uptake in the entire skeleton, the most prominent focus is seen in the right proximal femur (SUV 2.7). No PSMA expression was noted in the prostate or in lymph nodes. No sign of involvement of liver, lung, brain, or any other organ.
11FEB2020	Two years after 4 PRLT cycles, there is ongoing remission of the previously widespread metastatic disease in the axial and appendicular skeletal (persistent response to PSMA radioligand therapy according to molecular imaging criteria) with only very mild / negligible residual uptake. The primary tumor does not demonstrate any significant residual PSMA expression anymore. There is no evidence of PSMA positive lymph node, visceral or new metastases.
11FEB2019	Nearly complete remission of disease according to molecular imaging criteria; only very mild uptake noted in the central skeleton and sternum; no new and no intense PSMA-avid lesions noted.
14MAY2018	In comparison with previous Ga-68 PSMA PET studies, there is continued response seen after four PRLT cycles with further regression of disease and decrease of PSMA expression in previously existing extensive bone and bone marrow metastases; primary tumor located in dorsal left prostate lobe demonstrates only mild residual PSMA expression; no evidence of PSMA-positive lymph node, visceral, or new metastases.
15JAN2018	In comparison with previous Ga-68 PSMA PET studies, there is excellent response seen after three PRLT cycles with significant regression of disease and decreased PSMA expression in previously known bone and bone marrow metastases; primary tumor located in dorsal left prostate lobe demonstrates no significant change; no evidence of PSMA-positive lymph node or new metastases.
24JUL2017	In comparison with previous Ga-68 PSMA PET studies, there is excellent response seen after two PRLT cycles with significant regression of disease and decreased PSMA expression (ex. by 84% in the target lesion) in previously known bone and bone marrow metastases; primary tumor located in dorsal left prostate lobe shows a decrease of uptake by 44%; no evidence of PSMA-positive lymph node or new metastases.
07MAY2017	Intense but decreasing uptake – especially in the pelvis, in comparison with Lu-177 PSMA-617 post-therapy scan after first PRLT – was noted in the multiple PSMA-expressing bone metastases on scans obtained up to 46hr post-injection with an excellent target-to-background ratio, corresponding with molecular response to first PRLT therapy.
14MAR2017	Disseminated, intense PSMA-positive bone and bone marrow metastases present in the whole skeleton (skull, vertebral column, ribs, pelvis, and proximal extremities); primary tumor in the dorsal left prostate lobe; no evidence of PSMA positive lymph nodes. In comparison with prior DEC2016 Ga-68-PSMA PET/CT in Houston, there is progression of disease with increasing PSMA-expression in disseminated bone metastases and new osseous lesions (especially in pelvis).
30DEC2016	Multiple foci of increased Ga-68 PSMA uptake involving axial and appendicular skeleton consistent with multifocal active bone metastases. No evidence of metastasis to soft tissue of chest, abdomen, or pelvis.

## 3 Discussion

Prostate cancer is the fifth leading cause of death worldwide resulting in more than 350,000 deaths in 2018 alone (18–20). Additionally, mCRPC is generally incurable and fatal despite decades of research. While local or regional stages of prostate cancer have a nearly 100% five-year survival rate, the mCRPC five-year survival rate remains around 31%, demarcating a clear need for better diagnostic and therapeutic options (21).

The U.S. FDA approval of radiopharmaceutical ^177^Lu–PSMA-617 for the treatment of PSMA-positive mCRPC in March of 2022 marks an important advancement in mCRPC diagnostics and therapeutics. Approval follows the results of the VISION clinical trial (NCT03511664), where image-based progression-free survival was ~2.5 times longer when ^177^Lu–PSMA-617 was combined with standard care for adult patients with PSMA-positive mCRPC who had been treated with ADT and taxane-based chemotherapy (22). Similarly, the overall survival was four months longer, a 35% increase, when ^177^Lu–PSMA-617 was combined with standard care compared to standard care alone. These significant improvements in progression-free and overall survival for mCRPC patients demonstrate the powerful application of theranostics. While ^177^Lu–PSMA-617 in combination with abiraterone, the treatment course used in this patient, is not currently approved in the U.S., recent literature supports the potential for improved outcomes with this regimen (23).

Theranostics have shown great benefit in multiple settings, beginning with radioactive iodine for thyroid cancer in the 1940s, and more recent applications in gastroenteropancreatic neuroendocrine tumors (^177^lutetium DOTATATE), neuroblastomas (^123^I-metaiodobenzylguanidine), and melanoma (platinum nanoparticles, among others) (24–27). Combining imaging for better characterization, staging, and disease progression with targeted, localized radiation can decrease side effects and improve quality of life for specific patients. However, correctly identifying which patients will respond best remains a challenge. The use of PET/CT scanning in comparison with targeted radioligand imaging may help to highlight disease heterogeneity, providing more insight for patient eligibility.

For this patient, ^177^Lu–PSMA-617 was particularly successful likely at least in part due to the disease being limited to bony metastases and lymph nodes rather than soft tissue involvement. Interestingly, germline analysis in this patient had revealed a mutation in the *TSC2* gene. *TSC2* encodes the protein tuberin, which is involved in inhibiting mTOR signaling, a canonical pathway involved in cell growth and proliferation whose disfunction is related to oncogenic signaling, including in mCRPC (28, 29). Loss of function in *TSC2* results in increased mTOR activity (30). Additionally, the PI3K-mTOR signaling pathway is often dysregulated in prostate cancer (31). This pathway interacts with multiple signal transduction cascades, including the androgen receptor, MAPK, and WNT pathways, that can promote tumor growth (28). Moreover, mechanistic studies suggest there is a correlation between PSMA expression and increased mTOR activity (32). The possible role of downstream aberrant activity in mTOR signaling in the presence of this patient’s high PSMA expressivity may provide some insight into his notable response to this therapy.

In the absence of curative therapy, ^177^Lu–PSMA-617 represents a promising theranostic approach for prostate cancer, and potentially other PSMA-expressing cancers as well (33, 34). Recent clinical studies have underscored the utility of this theranostic option showing it is non-inferior to docetaxel (35), may have potential for use as an earlier line treatment in chemo-naïve mCRPC due to its efficacy and low toxicity (36), and can also be used in heavily pretreated patients with similar effects (37). The remarkable response of our patient highlights the potential of theranostics to monitor and control extensive disease with excellent tolerability and restoration of quality of life.

## 4 Patient perspective

I went for my annual physical with my primary care provider and found out that my PSA was 1800. I had scans and went to see an oncologist, and he told me I didn’t have much time - maybe 6 months to 1 year if I went on chemotherapy. So, I did (and lost my ponytail as a result), and responded pretty well at first, but then I got worse. My Private Health team told me about a new treatment available in Germany, so I went to see Dr. Baum, and that turned out quite well. After the first treatment, he showed me the scans and said the cancer had retreated a bit and he was encouraged. It got smaller and smaller with each subsequent treatment, and after the 4th treatment he couldn’t see any remaining disease. I go back every year for scans, but the disease looks like it is all gone. The treatments were not uncomfortable and didn’t hurt, and though the food at the clinic was terrible, the people were fun. I have kept in touch with one who also had a great response to the treatment.

## Data availability statement

The original contributions presented in the study are included in the article/supplementary material. Further inquiries can be directed to the corresponding authors.

## Ethics statement

Ethical review and approval was not required for the study on human participants in accordance with the local legislation and institutional requirements. The patients/participants provided their written informed consent to participate in this study. Written informed consent was obtained from the individual(s) for the publication of any potentially identifiable images or data included in this article.

## Author contributions

RB: Treated the patient. DP, JZ, AM, EL, EG: Managed the case. ML, TW, LA-E, EG: Drafted and edited the manuscript. All authors contributed to the article and approved the submitted version.
